# Crowded Out

**Published:** 1886-03

**Authors:** 


### CROWDED OUT.

A number of editorial articles are unavoidably left out to make room for the usual book notices. Among the postponed is the letter "To Junior Dentists," which will appear in the next number. Editorials are usually the first to go to the wall when there is a lack of space.

